# The Nutritional Cytokine Leptin Promotes NSCLC by Activating the PI3K/AKT and MAPK/ERK Pathways in NSCLC Cells in a Paracrine Manner

**DOI:** 10.1155/2019/2585743

**Published:** 2019-04-18

**Authors:** Fengzhou Li, Shilei Zhao, Tao Guo, Jinxiu Li, Chundong Gu

**Affiliations:** ^1^Department of Thoracic Surgery, The First Affiliated Hospital of Dalian Medical University, Dalian, Liaoning 116011, China; ^2^Lung Cancer Diagnosis and Treatment Center, Dalian 116011, Liaoning, China

## Abstract

**Purpose:**

Leptin is a nutritional cytokine encoded by the obesity gene whose concentration in the tumor microenvironment is closely related to the occurrence and progression of cancer. However, previous evidence has suggested that there is no clear relationship between serum leptin concentrations and lung cancer progression. Cancer-associated fibroblasts (CAFs), the most abundant component of the tumor microenvironment in a variety of solid tumors, were recently reported to produce leptin. Therefore, it was inferred that leptin is most likely to affect non-small-cell lung cancer (NSCLC) through an autocrine and paracrine mechanism. In the current study, we investigated the paracrine effect and mechanism of leptin produced by CAFs on NSCLC by establishing a novel in vitro cell coculture system.

**Methods:**

A noncontact coculture device was designed and made by 3D printing. CAFs and paired normal lung fibroblasts (NLFs) from 5 patients were successfully isolated and cocultured with two NSCLC cell lines in a coculture system. The background expression of leptin was detected by western blot. The in situ expression of leptin and its receptor (Ob-R) in NSCLC tissues and paired normal lung tissues was analyzed by immunohistochemistry. Furthermore, we downregulated the expression of leptin in CAFs and assessed changes in its promotion on NSCLC cells in the coculture system. Finally, changes in the phosphorylation of ERK1/2 and AKT were examined to investigate the molecular mechanisms responsible for the paracrine promotion of NSCLC cells by leptin.

**Results:**

Leptin was overexpressed in nearly all five primary CAF lines compared with its expression in paired NLFs. IHC staining showed that the expression of leptin was high in NSCLC cells, slightly lower in CAF, and negative in normal lung tissue. Ob-R was strongly expressed in NSCLC cells. The ability of A549 and H1299 cells to proliferate and migrate was enhanced by high leptin levels in both the cocultured fibroblasts and the culture medium. Furthermore, western blot assays suggested that the MAPK/ERK1/2 and PI3K/AKT signaling pathways were activated by leptin produced by CAFs, which demonstrated that the functions of paracrine leptin in NSCLC are as those of the serum leptin to other cancers.

**Conclusion:**

Leptin produced by CAF promotes proliferation and migration of NSCLC cells probably via PI3K/AKT and MAPK/ERK1/2 signaling pathways in a paracrine manner.

## 1. Introduction

Lung cancer is the most common malignancy worldwide [1]. Non-small-cell lung cancer (NSCLC) accounts for approximately 85% of all lung cancers [2], and is usually diagnosed at an advanced stage, often with metastases [3]. Therefore, the elucidation of the mechanisms involved in occurrence and progress of NSCLC could lead to novel diagnostic and therapeutic approaches [4].

Obesity is considered to be a growing health problem worldwide [5]. Leptin, a 16 kDa peptide hormone encoded by LEP gene (an obesity gene) and a nutritional cytokine, is closely related to cancer [6]. The main function of leptin is to regulate energy metabolism and fat synthesis in the body, and it also amplifies inflammation and immunity signals [7]. Leptin exerts its effects through a specific transmembrane receptor on the surface of its target cells, the obesity receptor (Ob-R), which is assigned to the class I cytokine receptor family [8]. The binding of leptin to its receptor activates multiple downstream signaling pathways such as those involving JAK2-STAT3, mitogen-activated protein kinase (MAPK), and phosphatidylinositol 3-kinase/ protein kinase B (PI3K/AKT) [9]. Therefore, leptin-mediated signaling pathways play an important role in cancer cell proliferation, invasion, and metastasis [10].

In recent years, crosstalk between the tumor microenvironment and cancer cells has received increasing attention [11]. The tumor microenvironment is the internal environment in which cancer cells intergrow and interact with the “nontumoral” components [12]. Cancer-associated fibroblasts (CAFs), the most abundant component of the tumor microenvironment of NSCLC, account for 70% of the total number of cells in solid tumors [13] and are most often identified by the expression of myofibroblastic biomarkers such as *α*-smooth muscle actin (*α*-SMA) [14]. Besides, fibroblast activating protein (FAP) and vimentin are also used as auxiliary markers to identify CAF [15]. CAFs can promote tumor progression through the secretion of various cytokines.

In addition to its synthesis by adipocytes, leptin can also be synthesized by epithelial cells or fibroblasts. Leptin is usually absent in nonneoplastic tissue; however, recent studies demonstrated that, leptin was also secreted by CAFs in breast cancer [16]. Thus, we inferred that CAF in NSCLC tissues can also produce leptin and affect the function of NSCLC in a paracrine manner. This hypothesis is supported by several recent pieces of evidence, as the increase of leptin concentration in the microenvironment was related to the occurrence and metastasis of several types of cancer [17–19]. On the other hand, a recent meta-analysis [20] suggested that there may be no clear relationship between the serum leptin concentration and lung cancer progression. This may be due to the special histological characteristics of alveoli and bronchi. Therefore, leptin is most likely to affect NSCLC through autocrine and paracrine effects. In the current study, we investigated the protumorigenic effect of leptin produced by CAFs on NSCLC by establishing a novel in vitro cell coculture system.

## 2. Methods

### 2.1. Cell Lines

Human embryonic lung fibroblasts (HLF) and the human NSCLC cell lines A549 and H1299 were obtained from the American type culture collection (ATCC), and all cell lines were cultured in DMEM (Gibco) supplemented with 10% fetal bovine serum (FBS, Gibco) and penicillin/streptomycin at 37°C in 5% CO_2_.

### 2.2. Culture of Primary Fibroblasts

Tissue samples from 5 patients who had a definitive pathological diagnosis of stage IIIA NSCLC were resected by thoracoscopic lobectomy in the 1st Hospital of Dalian Medical University in April 2018. The tissues were soaked in DMEM at 0°C and digested within 2 h of resection. Tumor tissues and their paired normal lung tissues (4~5 cm away from the incisal margin) were homogenized and digested for 2.5~4 h at 37°C in DMEM containing 0.1 mg/mL DNase I (Roche, Switzerland) and 1 mg/mL collagenase (Roche, Switzerland). The cells were filtered with a 75 *μ*m filter and then resuspended and plated with DMEM containing 1% penicillin/streptomycin and 15% FBS. The cultures were maintained at 37°C in 5% CO_2_. After 5 passages, fibroblast purity was tested by RT-qPCR. Then, CAFs were immortalized with SV40-large T antigen (EX-SV40T-Lv105, GeneCopoeia, USA) following the recommended protocol.

### 2.3. Noncontact Coculture System

**Figure 1:**
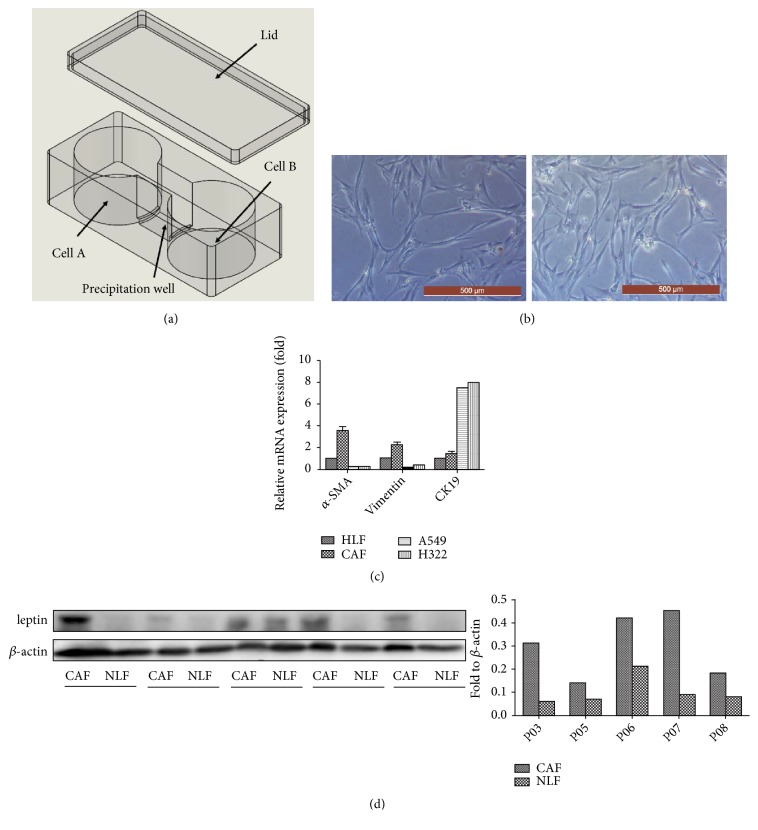
*Isolation and characterization of CAFs and NLFs.* (a) Three-dimensional model of the novel noncontact coculture device. (b) Morphology of primary fibroblasts under a microscope. Left: CAF, right: NLF. Scale bar = 500 *μ*m. (c) The purity of the primary cells was verified by RT-qPCR to determine the levels of *α*-SMA, vimentin, and CK19. (d) Leptin expression in primary CAF and NLF cell lines was detected with western blot. The quantification of leptin is shown in the bar chart.

A noncontact coculture device was designed (already in the patent examination and approval process in China) and made by 3D printing (Wanwan 3D, Dongguan, China) with highly transparent nontoxic resin to simulate the internal environment. The device was a vessel consisting of two culture wells and a precipitation well (Figure 1(a)). The culture wells and the precipitation well were separated by two 2 mm-high partitions. During cell seeding, the level of the liquid in both culture wells should be kept below the height of the partition. Fibroblasts and NSCLC cells were seeded into the two culture wells, at a 2:1 ratio. After cell adherence, DMEM containing 10% FBS was added to the vessel until the level was above the partition. Based on the design of the coculture device, floating cells do not go directly into the contralateral culture well but are deposited into the precipitation well. Before anything was done to the cells, the medium in the precipitation well was drained in case any floating cells entered their contralateral culture well. Then, the two cell lines were isolated again, and 0.5% trypsin was used for separate passage/collection of the two cell lines.

### 2.4. Conditioned Medium

Fibroblasts were cultured in DMEM with 10% FBS and routine antibiotics. Then, the medium was changed to FBS-free medium when the cells had grown to 80% confluence. After 48 h, the supernatant was extracted and centrifuged at 1600×g for 10 min. The conditioned medium (CM) was stored at –79°C and used for the colony formation assay.

### 2.5. Reagents and Antibodies

Recombinant human leptin was purchased from Pepro Tech (Rocky Hill, USA). A primary antibody against *α*-SMA was purchased from Abcam (Cambridge, UK). Primary antibodies against Ob-R were purchased from Wanleibio (Shenyang, China). A leptin antibody was obtained from Bioss (Beijing, China). Other primary antibodies against AKT, p-AKT, ERK, and p-ERK were obtained from Cell Signaling Technology (Massachusetts, USA). And other chemicals were purchased from Sigma (St. Louis, USA) and Vetec (St. Louis, USA).

### 2.6. Plasmids and Lentivirus

pLK0.1-PXR-shRNA targeting the LEP gene was acquired from the Institute of Cancer Stem Cell at Dalian Medical University and was packaged with the pMD2.G lentivirus packing system (GeneCopoeia, USA). Viral packaging was conducted based on the recommended protocols and previous articles.

### 2.7. RNA Extraction and Real-Time RT-PCR

Total RNA was extracted from CAFs and the NLFs using the TRIzol method according to previous articles [21]. TransScript One-step gDNA Removal and cDNA Synthesis Supermix (Transgene, Beijing, China) was used to synthesize first strand cDNA. SYBR Premix Ex Taq II (RR820A, Takara, Japan) was used for quantitative polymerase chain reaction according to the manufacturer's instructions. The primer sequences used were as follows: *α*-SMA:5'- ATTGCCGACCGAATGCAGA -3',5'- ATGGAGCCACCGATCCAGAC-3'; vimentin: 5'- TGCCGTTGAAGCTGCTAACTA -3', 5'- CCAGAGGGAGTGAATCCAGATTA -3'; CK-19: 5'- ACCAAGTTTGAGACGGAACAG -3', 5'- CCCTCAGCGTACTGATTTCCT -3'. The comparative Ct method was used to calculate relative changes in gene expression.

### 2.8. Immunohistochemistry Staining

IHC staining of leptin, Ob-R and *α*-SMA was performed according to the manufacturer's instructions. A streptavidin-peroxidase staining kit was purchased from ZSGB BIO (Beijing, China). The paraffin-embedded tissues were prepared by a pathology specialist, and dewaxed, and rehydrated in the lab. The experimental steps were carried out according to the instructions of the SP kit. The tissues were incubated with primary antibodies diluted to the recommended concentration overnight at 4°C with antibodies that was dilute to the recommended concentration. Then 3,3′-diaminobenzidine (DAB) staining was performed, and the results were observed under a microscope.

### 2.9. Western Blot Analysis

Total protein was collected according to the methods in our previous article [22]. The protein concentration of the cell lysis solution was assessed using a Thermo Fisher BCA kit. Then 30 *μ*g of total protein was run on 10% SDS-PAGE and transferred to a PVDF membrane. Samples were incubated with primary antibodies diluted to the recommended concentration overnight at 4°C. The samples were incubated with HRP-conjugated secondary antibody at room temperature for 2 h and protein bands were detected with a chemiluminescence device.

### 2.10. Colony Formation Assay

Briefly, A549 cells or H1299 cells were seeded into six-well plates (2 × 10^3^ per well) and incubated in complete DMEM for 24 h. Then, the medium was replaced by the abovementioned CM with 10% FBS, and the cells were cultured in a 37°C incubator with 5% CO2 for 2 weeks until they grew into macroscopic colonies. Finally, the medium was removed, and the cell colonies were stained with 0.1% crystal violet and counted.

### 2.11. Cell Migration Assay

Cell migration assays were performed in Transwell chambers with an 8.0*μ*m pore size (Corning, US). The cells were starved in FBS-free DMEM overnight before they were collected and resuspended. A total of 1×10^5^ A549 or H1299 cells were resuspended in 250 *μ*L DMEM with 10% FBS and injected into the upper chamber. A total of 3×10^5^ CAF cells were resuspended in 500 *μ*L DMEM with 10% FBS and injected into the lower chamber. After 24 h, the upper chamber was washed with PBS, the nonpenetrated cells were removed with a cotton swab, and the Transwell chamber was dried at RT. The migrated cells at the back side of the Transwell membrane were stained with 0.1% crystal violet and then observed under a microscope.

## 3. Results

### 3.1. Isolation and Characterization of CAFs and NLFs

CAFs and paired normal lung fibroblasts (NLFs) from 5 patients were successfully established, their cell morphology was first observed under an inverted microscope (Figure 1(b)), and no significant difference was observed between the two cell lines. The purity of the primary cells was verified by biomarkers using RT-qPCR. *α*-SMA, vimentin, and FAP were more strongly expressed, while CK19 and E-cadherin were more weakly expressed in the 5 CAF lines compared with their expression in the respective paired normal fibroblasts (Figure 1(c)). Leptin was overexpressed in almost all five primary CAF lines compared to its expression in paired corresponding NLFs (Figure 1(d)).

### 3.2. A Noncontact Coculture Model Was Successfully Established

One of the five CAF cell lines (CAF03), whose leptin background expression level was the highest, was selected for further study. Since it was difficult to stably culture NLFs in vitro, HLF cells were used as an alternative to the NLFs in the coculture system. In the coculture device, CAFs and HLF were successfully cocultured with A549 and H1299 cells through 5 consecutive passages.

### 3.3. Leptin Produced by CAFs Promotes the Proliferation of NSCLC

**Figure 2:**
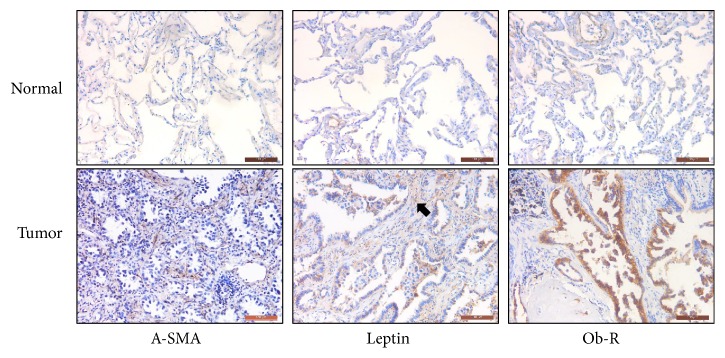
*Immunohistochemical analyses of α-SMA, leptin and Ob-R in NSCLC.* The expression of *α*-SMA, leptin, and Ob-R in the tumor and paired normal lung tissues of a 54-year-old male patient with acinar growth predominant lung adenocarcinoma was detected by IHC assay. The leptin overexpressed in the tumor stroma is marked with a black arrow in the corresponding position. Scale bar = 100 *μ*m.

**Figure 3:**
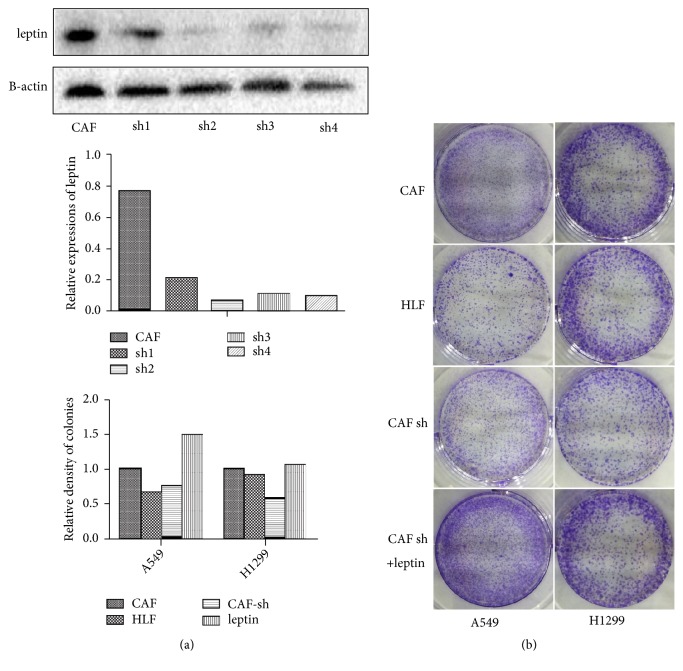
*The proliferation of NSCLC cells was inhibited by the downregulation of leptin in CAFs.* (a) The efficiency of gene intervention by four different shRNA fragments was verified by western blot. (b) The proliferation of NSCLC cell lines treated with the CM of different fibroblasts was assessed with a colony formation assay. The quantification of cell colonies is shown in the bar chart.

The original tumor tissue of the CAF03 cell line (from a 54-year-old female patient with stage IIIA acinar growth predominant lung adenocarcinoma) and its adjacent normal lung tissue were stained by immunohistochemistry, and *α*-SMA clearly marked the location of the stroma. IHC staining showed that leptin was highly expressed in epithelial cells and expressed at a slightly lower level in the cytoplasm of tumor stromal cells (mainly CAF). In contrast, both *α*-SMA and leptin were not expressed in normal lung tissues (Figure 2). Ob-R was strongly expressed in NSCLC cells and was expressed at low levels in both tumor stroma and normal tissues. Subsequently, the LEP gene in CAFs was stably downregulated by lentivirus transfection (sh4) with shRNA (Figure 3(a)). The ability of A549 and H1299 cells cultured with CAF-CM to form colonies was greater than that of cells cultured with HLF-CM. However, A549 and H1299 cells cultured with shLEP-CAF-CM showed inhibited proliferation, while this decrease in the ability to form colonies was reversed by the addition of 50 ng/ml recombinant leptin into the CM (Figure 3(b)).

### 3.4. Leptin Produced by CAFs Promotes Migration of NSCLC

**Figure 4:**
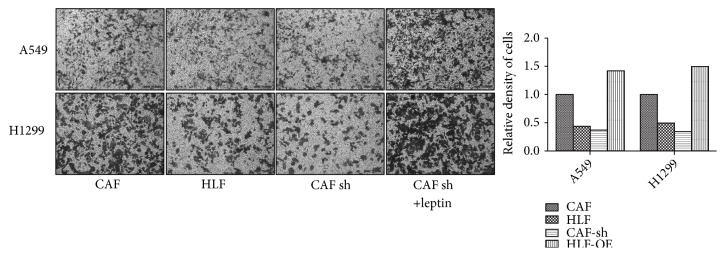
*The migration of NSCLC cells was inhibited by the downregulation of leptin in CAFs.* The migration of NSCLC cell lines cocultured with different fibroblasts was assessed by Transwell migration assay. The quantification of the migrated cells is shown in the bar chart.

Similar to the results of the cloning assay, the migration of A549 and H1299 cells cocultured with CAFs was enhanced compared with that of cells cocultured with HLF cells. While the expression of leptin was downregulated by shLEP in CAFs, the cocultured A549 and H1299 cells showed inhibited migration. However, this decrease in migration was reversed by the addition of 50 ng/ml recombinant into the medium (Figure 4).

### 3.5. Leptin Produced by CAFs Mediates the Activation of Inflammatory Cytokine-Related Pathways in NSCLC

**Figure 5:**
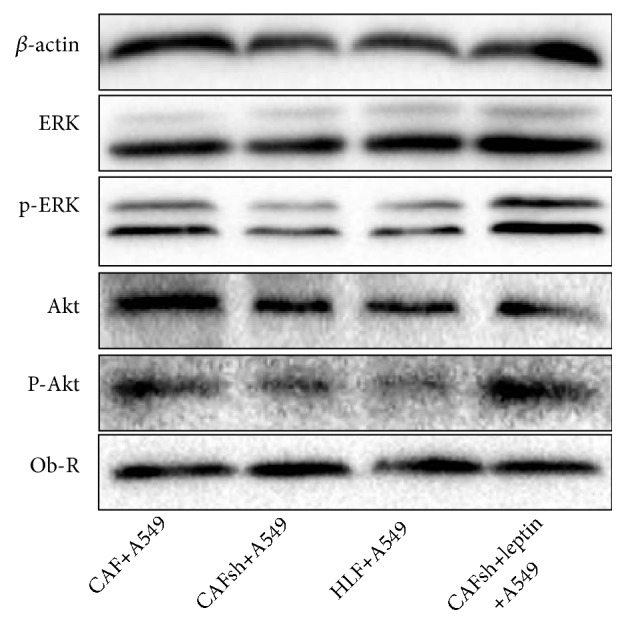
*The phosphorylation of ERK1/2 and AKT depends on paracrine leptin.* (a) Leptin significantly increased the phosphorylation of ERK1/2 and AKT. Three independent experiments were performed, and representative protein bands from one of the three tests are shown. Leptin treatment: 50 ng/ml, 1 hour.

A549 cells grown with HLF, CAFs, CAF-shLEP, and CAF-shLEP+leptin were continuously cultured to four passages in the coculture system. Then the total proteins of the A549 cells were analyzed by western blot (Figure 5). The expression of Ob-Rb was no different in the four groups. Furthermore, changes in the phosphorylation of ERK1/2 and AKT were examined to investigate the molecular mechanisms responsible for the paracrine promotion of NSCLC cells by leptin. Similarly, leptin significantly increased the phosphorylation of ERK1/2 and AKT. This result suggested that these two pathways, which are closely related to the internal inflammatory malignant environment, were activated under conditions of high paracrine leptin secretion.

## 4. Discussion

CAFs act on NSCLC cells by secreting a variety of cytokines, thereby affecting the proliferation, migration, invasion, and other biological functions of NSCLC cells. At the same time, cancer cells have positive feedback on the activation and maintenance of the cancer-associated properties of CAFs [23]. Researchers have tried many methods to simulate tumor microenvironment in both in vivo and in vitro experiments, especially to revive the interaction between CAFs and tumor cells. Classic models include conditioned medium, the Transwell system [24], the Boyden chamber system [25], and xenoanimal coinjection models [26]. In recent years, microfluidic chip technology [27] and the patient-derived xenograft mouse model [28] (PDX) have also become common research tools. In the current study, we introduced a novel noncontact coculture device, that solves the limitations of previous models in terms of cell quantity and passages, and its practicality was experimentally verified.

Leptin is a cytokine and hormone first shown to be secreted by adipose tissue [29]. Leptin is also produced and secreted by many nonadipose tissues, including gastric mucosal [30], breast [31], muscle [32], placenta [33], and lung tissues [34]. Studies have suggested that a leptin-enriched microenvironment promotes tumor cells [17–19, 35]. In the current study, we confirmed that leptin was overexpressed in NSCLC and was produced not only from epithelial cells, but also from CAFs. Furthermore, we found that the receptor of leptin, Ob-Rb, was only overexpressed in NSCLC cells, and was not expressed in CAFs. As previous studies have shown that serum leptin has no significant effect on lung cancer, we hypothesized that leptin most likely affects NSCLC through autocrine and paracrine mechanisms.

Furthermore, we observed the effect of paracrine leptin on NSCLC, and found that CAF-produced leptin reversibly promoted colony formation and migration of NSCLC cells. On this basis we found that the function of paracrine leptin was similar to that of the addition of recombinant leptin directly into the medium. Nevertheless, the downregulation of leptin produced by CAFs can offset this effect. This result confirmed that paracrine leptin promotes the proliferation and migration of NSCLC cells. However, given that NSCLC cells themselves also produce large amounts of leptin, whether autocrine leptin has an effect on NSCLC still needs to be explored in future studies.

The function of leptin depends on a specific receptor (leptin receptor, OB- Rb) on the surface of its target cells [36]. Ob-R is expressed at very low levels in epithelial cells from normal human mammary glands, but it is overexpressed in cancer cells [37]. A variety of inflammatory cytokines and inflammatory-mediating pathways are downstream of the leptin-OB-Rb signaling pathway, such as the PI3K/AKT, MAPK/ERK1/2, JAK2/STAT3, and insulin receptor substance (IRS) pathways [38]. In the current study, we investigated changes in key molecules from several signaling pathways in NSCLC cells cocultured with CAFs and found that the MAPK/ERK1/2 and PI3K/AKT signaling pathways were activated by leptin produced by CAFs, which demonstrated that the functions of paracrine leptin in NSCLC were similar to those in the same pattern with that of the serum leptin to in other cancers. Thus, it was concluded that leptin produced by CAFs promotes the proliferation and migration of NSCLC cells probably via the PI3K/AKT and MAPK/ERK1/2 signaling pathways in a paracrine manner.

## Data Availability

The experimental data used to support the findings of this study are included within the article. The 3D printing drawing data are available from the corresponding author upon request. Detailed data of the patients involved in the study are restricted by the Ethics Committee of the 1st Hospital of Dalian Medical University. Data are available from Dr. Gu (guchundong@dmu.edu.cn) for researchers who meet the criteria for access to confidential data.
